# Stress reduction strategies in breast cancer: review of pharmacologic and non-pharmacologic based strategies

**DOI:** 10.1007/s00281-020-00815-y

**Published:** 2020-09-18

**Authors:** Rohit Gosain, Elizabeth Gage-Bouchard, Christine Ambrosone, Elizabeth Repasky, Shipra Gandhi

**Affiliations:** 1 Department of Medicine, Roswell Park Comprehensive Cancer Center, Buffalo, NY USA; 2 Department of Medicine, UPMC Hillman Cancer Center, Chautauqua, NY USA; 3 Department of Cancer Prevention and Control, Roswell Park Comprehensive Cancer Center, Buffalo, NY USA; 4 Department of Immunology, Roswell Park Comprehensive Cancer Center, Buffalo, NY USA

**Keywords:** Breast cancer, Stress, Complementary and alternative medicine, β-Blocker, Yoga, Pharmacological, Non-pharmacological strategies, Fatigue, Anxiety, Quality of life

## Abstract

Breast cancer is the most common cancer diagnosed in women. It is associated with multiple symptoms in both patients and caregivers, such as stress, anxiety, depression, sleep disturbance, and fatigue. Stress appears to promote cancer progression via activation of the sympathetic nervous system releasing epinephrine and norepinephrine as well as activation of hypothalamic-pituitary-adrenal axis releasing cortisol. These stress hormones have been shown to promote the proliferation of cancer cells. This review focuses on stress-reducing strategies which may decrease cancer progression by abrogating these pathways, with a main focus on the β-adrenergic signaling pathway. Patients utilize both non-pharmacologic and pharmacologic strategies to reduce stress. Non-pharmacologic stress-reduction strategies include complementary and alternative medicine techniques, such as meditation, yoga, acupuncture, exercise, use of natural products, support groups and psychology counseling, herbal compounds, and multivitamins. Pharmacologic strategies include abrogating the β2-adrenergic receptor signaling pathway to antagonize epinephrine and norepinephrine action on tumor and immune cells. β-Blocker drugs may play a role in weakening the pro-migratory and pro-metastatic effects induced by stress hormones in cancer and strengthening the anti-tumor immune response. Preclinical models have shown that non-selective β1/2-blocker use is associated with a decrease in tumor growth and metastases and clinical studies have suggested their positive impact on decreasing breast cancer recurrence and mortality. Thus, non-pharmacological approaches, along with pharmacological therapies part of clinical trials are available to cancer patients to reduce stress, and have promise to break the cycle of cancer and stress.

## Introduction

Despite advancements in the field of cancer, from diagnosis and treatment standpoints, breast cancer remains a major health concern [1]. Stress appears to be an important factor affecting the quality of life of both patients and their caregivers. This review sheds light on how stress perceived by patients when they are diagnosed with cancer plays a role in cancer progression. Additionally, this review also highlights important research being conducted on non-pharmacological and pharmacological strategies for stress reduction in breast cancer patients. Breast cancer affects approximately 12% women in the USA during their lifetime [2]. The incidence rate for breast cancer is increasing globally, with 1.38 million new cases diagnosed in 2008, to about 1.7 million reported in 2012 [3–5] and 2.1 million cases in 2018 [6]. A significant proportion of this rise in incidence is attributed to changes in environmental and lifestyle factors [7].

Breast cancer is associated with symptoms which are physical, psychological, and cognitive [8]. The psychological consequences from breast cancer diagnosis include stress, anxiety, depression, and impaired cognitive function [9–12]. Physical symptoms that impact breast cancer patients range from pain, sleep disturbances, to fatigue [13–15]. Fatigue, which results in lack of energy, affects 40–80% of patients during and after treatment [16]. These symptoms which impact patients, such as fatigue, anxiety, and depression remain and/or occur years after the treatment, which significantly diminish quality of life and contribute to stress [17, 18].

A breast cancer diagnosis can also have profound consequences for patients’ caregivers. Informal cancer caregivers are defined by NCI as individuals (commonly family members) who provide cancer care or other supportive activities that is typically uncompensated and provided at home [19]. Being a caregiver to a cancer patient can include multiple role demands and stressors [20]. Caregivers can experience even more distress, anxiety, and depressive symptoms than cancer patients, and over half of all cancer caregivers experience clinically significant distress [21, 22]. Caregivers with greater psychological stress are more likely to binge drink, smoke, avoid exercise, and feel physically unwell, have lower self-rated health and experience health declines and impaired immune function [22–25]. Caregivers with higher stress experience more difficulty executing caregiving tasks [19, 23, 26, 27]. At the same time, caregivers with a lower stress management ability report less preparedness and decision-making efficacy [28]. Recent research has begun to examine how the physical and mental health outcomes of patients and caregivers are related; increased caregiver stress is associated with lower patient perceived quality of cancer care [23, 27, 29]. There are dyadic relationships between stress experiences among breast cancer patients and their caregivers. For example, increased caregiver stress is associated with patient depression and anxiety [30].

To deal with these symptoms, patients and their caregivers often explore different coping mechanisms, ranging from non-pharmacologic means like meditation, yoga, and exercise to pharmacologic modalities. This review focuses on use of these modalities among breast cancer patients to manage stress associated with their cancer diagnosis. A total of 48 to 80% of American women diagnosed with breast cancer make use of complementary therapies, while worldwide it ranges from 33 to 47% [31]. For example, meditation is one complementary modality which helps patients cope with pain, stress, depression and adverse effects [32, 33]. Meditation has also been shown to improve sleep quality, feelings of empowerment, competence, personal growth, sense of calm, serenity, and balance [15, 34]. In addition to non-pharmacological remedies, pharmacological agents have also been used. The human body’s natural reaction to acute psychosocial stress includes “fight or flight” response, resulting in activation of sympathetic nervous system [35]. Norepinephrine (NE) as a driver of the sympathetic response has been examined as a possible contributor to cancer progression [36–38]. β-Adrenergic receptors have been shown to be responsible for cancer development, progression, and angiogenesis [39, 40]. Multiple preclinical studies in various cancers have documented that β-blockers can arrest the stress-induced catecholamine release of epinephrine and NE, resulting in suppression of pro-growth and pro-angiogenic effects [41–44] and improve anti-tumor immunity [45–48].

The diagnosis of cancer can be associated with physical and psychological stress in patients. Stress has been shown to promote cancer growth. As a result, to counter this stress response, patients utilize several non-pharmacological strategies. This review describes the mechanism of stress response in cancer, and additionally highlights the pharmacologic and non-phamacologic approaches utilized by cancer patients focusing on inhibiting the elevated stress-induced sympathetic nervous system. These approaches enhance quality of life and also help cope with stress which may result in better survival outcomes.

## Stress promotes cancer progression

The term “stress” captures the psychophysiological processes a person may have in response to an event perceived to be harmful or challenging [49]. When focusing on physiologic responses to stress, it is important to understand the mechanism behind the stress response which may further promote cancer progression. Two branches of the nervous system are involved in stress response, the sympathetic nervous system (SNS), and the hypothalamic-pituitary-adrenal (HPA) axis. These pathways are activated when stress is perceived and result in release of several neurotransmitters and hormones that facilitate behavioral and biochemical changes thereafter [48]. In cancer patients, it is suspected that this dysregulation of the SNS and HPA axis is responsible for cancer progression [50].

### HPA axis and cortisol in cancer cells

The principal effectors of the stress response are located in the paraventricular nucleus (PVN) of the hypothalamus, the anterior lobe of the pituitary gland, and the adrenal gland. This collection of structures is referred to as the HPA axis. In response to stress, corticotropin-releasing factor (CRF) released from the hypothalamus acts on the pituitary gland to induce the release of adrenocorticotropic hormone (ACTH) into the systemic circulation. The principal target for circulating ACTH is the adrenal cortex, where it stimulates glucocorticoid synthesis and secretion from the zona fasciculata [51]. Glucocorticoids, such as dexamethasone, stimulate anti-apoptotic gene expression and antagonize the cell death mechanism resulting in progression of epithelial tumors [50]. Psychological stressors, acute or chronic, have been shown to disrupt the neuroendocrine circadian rhythm which may promote cancer growth [52]. This is exemplified in a study of patients with metastatic breast cancer where a hyperactive adrenal gland resulted in chronically elevated resting plasma cortisol levels, along with possible tamoxifen administration resulting in higher cortisol. Interestingly, a linear relationship was observed between cortisol levels and the cancer stage, where patients with metastatic breast cancer had significantly higher levels of basal cortisol compared to early stage breast cancer patients who had lower cortisol levels [53]. This increase in cortisol levels is concerning as it has been well documented that cortisol promotes cancer progression via direct activity on tumor cells by activating glucocorticoid receptor (GR) signaling pathway, and upregulating downstream anti-apoptotic genes such as serum/ glucocorticoid-regulated kinase 1 (*SGK1*) and mitogen activated protein kinase phosphatase 1 (*MKP1*)/dual specificity phosphatase 1 (*DUSP1*) [54]. At the same time, cortisol suppresses immune function, with decreases in natural killer (NK) cell activity and T cell proliferation [55].

### Sympathetic innervation and epinephrine/norepinephrine release in cancer cells

Release and function of catecholamines (norepinephrine and epinephrine) from the autonomic nervous system is best understood from the “fight-or-flight” response, also referred to as the acute stress response (physiological and psychological response from body in a stressful situation). This state of stress results in rapid heart rate and breathing, pale and flushed skin, dilated pupils, and trembling [56]. The adrenergic receptors, which are the target of epinephrine and norepinephrine, are responsible for regulating apoptosis, proliferation, and angiogenesis in normal tissues [48]. Adrenergic receptors are also expressed on various cancer cells, including breast, melanoma, pituitary, pancreatic, lung, melanoma and prostate cancer [57–63]. Studies have focused on neuroendocrine regulation of breast cancer progression via sympathetic nervous system with release of epinephrine and norepinephrine neurotransmitters [37, 38, 64–66]. Preclinical studies have further demonstrated that these neurotransmitters induce tumor cell invasion and migration aiding in metastasis [67–71]. Specifically in breast cancer, in vivo studies have demonstrated that adrenergic signaling has an association with increased nodal involvement and development of metastasis [72]. Kamiya et al. showed that breast cancer growth and progression can be stimulated with sympathetic nerve innervation in mice. Conversely, this growth can be suppressed with parasympathetic stimulation. Higher parasympathetic activity promotes energy conservation, while increased sympathetic activity depletes those energy stores [73]. This combination of elevated sympathetic nervous system activity along with parasympathetic underactivity has been associated with increased cancer related fatigue, activation of proinflammatory cytokine network, and an increased incidence of cancer recurrence in breast cancer [74–76].

In summary, stress promotes cancer progression via release of several hormones and neurotransmitters causing tumor cell progression and also inducing immune system dysfunction. To decrease stress, there are several non-pharmacological and pharmacological stress reduction strategies utilized by patients and their families.

## Non-pharmacological strategies to reduce stress

There are many complementary and integrative therapies employed by patients and their families to reduce stress and promote better quality of life, which as highlighted above, may provide significant anti-cancer effects. The National Center for Complementary and Alternative Medicine (NCCAM) is an initiative by the National Institutes of Health (NIH), comprised of diverse heathcare systems and practices [77]. Some of the modalities (part of NCCAM) include mind-body medicine, nutritional supplementation, use of herbal products, exercises, and other energy-based techniques.

Millions of Americans use some form of CAM, and this number has been growing each year. According to a survey conducted in 2007, about 38% of American adults and approximately 12% of American children used some form of CAM [78]. These numbers are significantly higher when focusing on breast cancer patients [79–83]. A European study, which included a total of 11 countries, reported 44.7% prevalence of CAM use in breast cancer patients [82]. Recent studies have demonstrated higher number of breast cancer patients using CAM to enhance their quality of life; with reports of 62.9% use in Germany, 81.9% in Canada, and 86.1% in the USA [79–81]. Among 86.1% patients in the US study who used CAM following breast cancer diagnosis, 47.5% used botanical supplements, 47.2% used other natural products, 28.8% used special diets, 64.2% used mind-body healing, and 26.5% used body/energy/other treatments [81]. Other CAMs used by breast cancer patients include spiritual approaches, such as prayers, meditation, and mental healing [84]. Spiritual and religious practices, especially praying, has been one of the coping strategies used by breast cancer patients [85]. In addition, manipulative and body-based methods such as therapeutic massage in breast cancer patients shows beneficial effects on the neuroendocrine and immune system, with reduction in anxiety levels, depression, anger, fear, and stress, and improvement in NK and lymphocyte counts [86].

### Mindfulness-based interventions

Mindfulness-based approaches in oncology acknowledge suffering as a normal part of cancer and cultivate acceptance of experiences to promote greater flexibility in coping with stress. Mindfulness emphasizes intentional focus on moment-to-moment experiences in a non-judgmental way [87]. Mindfulness interventions commonly focus on developing greater self-awareness, lowering reactivity in stressful situations, and mindful communication skills [87]. In cancer, mindfulness-based interventions have been shown to help patients cope with their feelings of loss of control, future uncertainty, and fears of recurrence that are associated with cancer diagnosis, treatment, and survivorship, while also providing psychological and overall-health-related benefits [88–90]. For example, one study of an 8-week mindfulness-based stress reduction (MBSR) program, including forty-nine breast cancer patients and ten prostate cancer patients, reported enhancement in quality of life, with decreased stress symptoms. At the same time, decrease in daily cortisol levels and Th1 (pro-inflammatory) cytokine levels, such as IFN-λ, TNF, IL-4, and IL-10, have been observed [91]. A number of recent meta-analyses have found that mindfulness-based interventions improved cancer patients’ stress, anxiety, and depression [92–94]. Randomized trials also show the efficacy of mindfulness-based interventions to improve cancer patients’ fatigue, insomnia, quality of life, psychological, behavioral, physiological, and biological markers of health [93, 95–98]. Many studies have investigated the use of holistic yoga (where yoga sessions varied, ranging from one to three times a week, each session lasting from 60 to 90 min) in breast cancer patients, and showed statistically significant benefits with improvement in depression, stress, anxiety, physical well-being, quality of life, fatigue, cancer pain, cognitive function, and appetite, and an increased tolerance to chemotherapy side effects, though no essential survival benefit has been reported [99–111]. However, important questions remain related to the relative efficacy of mindfulness-based approaches compared to other stress reduction strategies, the appropriate dose and timing of mindfulness-based interventions, participant characteristics that shape intervention acceptability and efficacy, and the mechanisms through which mindfulness-based interventions impact stress.

### Acupuncture

Multiple cancer- and treatment-related symptoms, such as depression, anxiety, hot flashes, neuropathy, nausea, vomiting, and radiation-induced xerostomia, result in stressful situations for breast cancer patients. Many breast cancer patients experience treatment-induced hot flashes and menopausal symptoms and instead of relying on medications, some seek other non-medical options. Acupuncture is one of the techniques that have shown advantages in distressed healthy adults [112]. A literature review reported that acupuncture enhances immune function through NK cell activity [113], based on stimulation of the acupoints, also known as “immuno-enhancing acupoint,” thus enhancing nitric oxide production which promotes secretion of β-endorphin [113, 114]. This peptide further reaches different locations of the body, and binds to opioid receptors expressed on surface of NK cells, which stimulates NK cells to release interferon-gamma (IFN- γ), further inducing expression of NK cell receptors and secretion of cytokines from other immune cells, promoting anti-carcinogenic immune functions [113–116]. Additionally, acupuncture may help breast cancer patients with menopausal symptoms via altering the autonomic nervous system [117]. Freedman et al. reported that hot flashes mainly occur because of sympathetic nervous system upregulation [118]. Acupuncture results in upregulation of parasympathetic nervous system, thus relieving symptoms of menopause [117]. Similarly, acupuncture has benefited cancer patients with vasomotor symptoms, anxiety, cancer-related pain, nausea, and vomiting [119–121]. By helping breast cancer patients deal with the above symptoms, acupuncture plays a role in improving stress levels in these patients. Though there is a benefit of utilizing acupuncture to help with quality of life measures in cancer patients, no survival benefit has been reported to date.

### Natural products

The use of herbal and natural products has increased over the past decade. These products are used by patients to manage treatment-related symptoms, and also to help with psychological stress post diagnosis [122, 123]. A study reported that the most frequently used CAM were vitamins (70%) and herbal products (26%) [124]. These numbers could be underestimated as well, as studies reveal that approximately 46 to 60% of all cancer patients do not tell their healthcare provider about the use of CAM therapy [125, 126]. Though patients take natural products and herbs to enhance their quality of life and for symptomatic management, there is little documented benefit of these products [122, 123]. Since cancer is an inflammatory state resulting in oxidative stress with increased production of pro-inflammatory cytokines, reactive oxygen species, cyclooxygenase (COX-2), nuclear factor κB (NFκB), some of the herbal products like curcumin, *Ashwagandha* (*Withania somnifera*) exert immunomodulatory activity by interacting with these key mediators and exert antitumor effects [127, 128]. A randomized clinical trial in subjects with history of chronic perceived stress using high concentration full-spectrum *Ashwagandha* root extract showed decrease in serum cortisol and improved resistance towards perceived stress and improved self-assessed quality of life [129]. Another prospective study in breast cancer patients showed that *Ashwagandha* resulted in improvement in cancer-related fatigue and quality of life, both of which indirectly result in better management of stress symptoms associated with breast cancer diagnosis and treatment [130]. Other commonly used natural and herbal products are, blue skullcap, gotu kola, guarana, kava, keenmind, lemon grass, passion flower and valerian. Kava when compared to others in the randomized controlled studies, showed benefit as an anxiolytic, while others showed no benefit [122]. However, use of kava has been associated with liver failure [122, 123, 131] and additional research is warranted.

A number of studies have shown benefits with vitamin and mineral supplementation, including improvement in stress, anxiety, and fatigue. There is evidence that these supplements could enhance antidepressant effects [132, 133], and vitamin B levels, namely folic acid (B9), pyridoxine (B6), and cobalamins (B12), have been shown to be lower in patients under psychological stress [133–136]. One might then posit that supplementation may help deal with the stress symptoms.

However, the use of dietary supplements may also have negative effects on treatment outcomes. In this era where the use of herbal and natural products is on a rise, and without results from rigorous trials at hand, it is important to inquire about the use of these products from every patient. There has been concern that use of antioxidants, herbs and vitamins can interact with chemotherapy, radiotherapy, and hormonal treatment. Such use of CAM in combination with other therapies has been reported to cause severe side effects, and at other times negatively impacting the treatment outcomes [137, 138]. Recently, we reported results from an ancillary study conducted in the context of a SWOG clinical trial for high-risk breast cancer [139]. In that study, patients reported on use of supplements before and during chemotherapy. Results showed that use of any antioxidant (vitamins C, E, and A, carotenoids, coenzyme Q10) during treatment was associated with a 40% increase in hazard of recurrence as well as death. Use of iron supplements before and during chemotherapy, as well as use of vitamin B12, was associated with a twofold increase in hazard of death. Although use of supplements was self-reported, this study of more than 1300 breast cancer patients suggests that use of dietary supplements, particularly during chemotherapy, should be carefully considered by patients and their clinicians.

Given the current data, while consumption of some of the natural products may help manage stressful symptoms associated with breast cancer diagnosis and enhance quality of life; their use may not always be safe given increased toxicities and decreased survival reported recently [137, 138]. Hence, use of this approach to help alleviate symptoms should be considered with caution.

### Support groups and psychology counseling

Dealing with a diagnosis of cancer can be difficult for patients. Connection with others experiencing cancer, such as through in-person or online support groups, may provide social and emotional support for some patients. Spiegel et al. reported that support group therapy in women with metastatic breast cancer resulted in a mean of 18 months longer survival in comparison to women in the control group [140–142]. This survival benefit through these support groups was later challenged by two randomized trials in breast cancer patients [143, 144]. Though these trials did not show that group therapy resulted in prolonged survival, they showed transient positive effects on mood and self-esteem, which indirectly resulted in improved stress levels [143, 144]. Results of other similar studies in different cancer settings have been inconsistent and no firm conclusions could be reached from a survival standpoint [145, 146].

Increasing emotional support, mood stability, and reducing isolation are key factors to enhance quality of life and well-being. Therefore, when a patient is diagnosed with breast cancer and struggling with coping with multiple stressors associated with the diagnosis, it is important to have appropriate counseling. Multiple studies have demonstrated that psychology counseling sessions, which can be either in-person or through tele-medicine, have shown improvement in health-related quality of life in all subscales. Kim et al. reported that psychological intervention showed significant improvement in fatigue, depression, diet, and readiness to exercise in breast cancer patients [147]. Other trials offering psychological counseling demonstrated reductions in depression and improvement in social and spiritual well-being, ultimately helping to deal with and manage stress [148–150].

### Exercise

In cancer patients, malignancy is a major source of fatigue, and treatment often adds significantly to stress. Patients are often counseled on the best ways to combat fatigue and stress, and exercise is a commonly recommended strategy to reduce these symptoms. For example, a study by Midtgaard et al. showed that patients receiving chemotherapy had improvement in psychological stress with an exercise regimen [151]. A study focusing on breast cancer patients receiving adjuvant chemotherapy showed that patients in the exercise group were found to have lower symptom severity scores and mood disturbance when compared to the non-exercise group patient population [152]. When focusing on early stage breast cancer, a study reported that patients who participated in a walking exercise program during radiation treatment had improvement in their fatigue, anxiety and sleep quality [153].

As discussed above, there is autonomic nervous system imbalance that can occur in cancer patients. The parasympathetic nervous system is responsible for vagal tone, which further accounts for heart rate variability (HRV) [154]. However, healthy adults, with lower HRV report more fatigue due to parasympathetic underactivity [155]. Similar results of low HRV have been observed in breast cancer survivors, which results in increased fatigue [156–158]. Exercise training and cardiopulmonary fitness result in increased HRV [159–161]. This is also evident in cancer patients, where a 16-week exercise intervention during and after treatment resulted in an improvement in HRV, demonstrating that exercise is a modality that helps restore the existing autonomic nervous system imbalance in cancer patients [162].

Preclinical studies in 4T1 mammary carcinoma, a mouse model of triple-negative breast cancer (TNBC), have shown that exercise slowed tumor progression and reduced the tumor-induced accumulation of myeloid-derived suppressor cells (MDSCs) with a relative increase in natural killer (NK) and CD8^+^T cell activation, resulting in a favorable immune environment [163]. Similar findings of exercise intervention in breast cancer patients with increased number and effector function of monocytes, macrophages, and NK cells have been observed [164]. Given the immune-enhancing ability of exercise interventions, the benefits have not just been limited to quality of life enhancement, but patients who exercise have also shown to have better survival outcomes. A recent study of newly diagnosed women with breast cancer in Australia randomized patients to either 8-month exercise intervention or usual care after 6 weeks postsurgery. After a median follow-up of 8.3 years, there were 11 (5.3%) deaths reported in the exercise group in comparison to 15 (11.5%) deaths in usual care group, with hazard ratio (HR) of 0.45 (95% CI 0.20–0.97) [165]. In a meta-analysis including 6 cohort studies with breast cancer patients, a HR of 0.66 (95% CI 0.57–0.77) for breast cancer specific mortality and 0.59 (95% CI 0.53–0.65) for overall survival (OS) was observed among patients involved in physical activity compared to patients with sedentary lifestyle [166]. Other studies have confirmed these findings [167]. Analysis of data from 5807 patients enrolled in the Roswell Park Comprehensive Cancer Center Shared Resource, the Data Bank and BioRepository, showed that habitual physical activity was associated with enhanced survival among patients with a number of cancers, including bladder, breast, colorectal, esophageal, prostate, skin, endometrial (uterine), and ovarian cancers [168]. Importantly, there was reduced risk among patients who had been physically inactive prior to diagnosis but began exercising after learning of their cancer. This beneficial effect of exercise on breast cancer could be due to decreased lifetime estrogen exposure, augmented immune function, lower body fat resulting in decrease in insulin resistance [166], or through other mechanisms. In the same ancillary study in the SWOG trial that assessed outcomes in relation to use of dietary supplement, we recently also queried patients about their recreational physical activity before, during, and after chemotherapy. In that study, patients who were meeting the Physical Activity Guidelines before and 1 year after diagnosis had significantly reduced hazard of recurrence (HR = 0.59, 95% CI 0.42–0.82) and mortality (HR = 0.51, 95% CI 0.34–0.77) compared to inactive patients [169].

Thus, exercise before, during, and after treatment in breast cancer patients does not only enhance quality of life, reducing stress and anxiety, but also improves overall survival, and therefore, should be employed in clinical practice.

## Pharmacological management to suppress stress-induced β-AR signaling pathway

Non-pharmacological strategies enhance quality of life by improving several symptoms, such as anxiety, stress, hot flashes, and fatigue in breast cancer patients as discussed above. However, there is limited data about a survival benefit with this approach. At the same time, it is challenging for patients battling cancer, who are dealing with physical and psychological stressors associated with the diagnosis, to further motivate themselves to indulge in these non-pharmacological strategies. As a result, in addition to non-pharmacological approaches, the use of pharmacological means has been explored in breast cancer patients to manage stress and the vicious cycle of adverse events triggered by stress.

Multiple prior studies have investigated the role of the β-adrenergic receptor signaling pathway in promoting breast cancer progression and role of pharmacologic approaches to inhibit this pathway. Another stress signaling pathway is the HPA axis as discussed before which promotes breast cancer cell growth, especially TNBC by acting on GR on the tumor cell. The role of GR signaling in breast cancer is evolving as data shows that dexamethasone administration in preclinical TNBC models can lead to cancer progression. This study showed that increase in stress hormones during breast cancer progression (cortisol, corticosterone, and ACTH) results in activation of the GR at distant metastatic sites, increased colonization, and reduced survival [170]. This knowledge is of utmost importance, particularly in the clinics, where glucocorticoids are used as anti-emetic and anti-inflammatory agents. With the recent approval of checkpoint inhibitors in TNBC, the use of glucocorticoids will further increase in breast cancer for the management of immune-related adverse events from checkpoint inhibitors [171]. Hence, data shows that glucocorticoids should be cautiously used in TNBC. In addition, these findings also highlight the importance of better stress management to abrogate this signaling pathway in breast cancer patients where higher cortisol levels have been observed [53]. Over the last few years, studies have shown that GR signaling in TNBC results in worse relapse-free survival, but its correlation with stress perception has not been investigated [172]. This interesting interaction of the stress pathway involving HPA axis, GR and its signaling is evolving and further study of this area is warranted to develop approaches to target this pathway to improve outcomes [173]. Preclinical studies have shown that GR antagonists can overcome chemotherapy resistance in TNBC [174]. Since GR is highly expressed in TNBC, clinical trials are actively investigating if GR antagonists such as mifepristone and CORT125134 in combination with chemotherapy could help improve survival of GR-positive TNBC [175]. A detailed discussion of the GR signaling pathway is beyond the scope of this review.

This article focuses on discussion of the stress-induced β-AR signaling pathway in breast cancer patients and highlights both preclinical and clinical studies targeting this pathway to inhibit cancer growth and improve survival outcomes.

### Preclinical studies using pharmacologic blockade

Stress results in activation of the SNS which releases neurotransmitters epinephrine and NE that act on β-adrenergic receptors. As discussed, the β-adrenergic signaling pathway plays a key role in tumor progression via direct pro-tumorigenic effect on cancer cells and inhibition of anti-cancer immunity. To combat these mechanisms, several preclinical studies have explored the role of repurposing β-blockers as an effective anti-tumor armamentarium since it is relatively inexpensive and safe. Both selective (metoprolol, atenolol) and non-selective β-blockers (propranolol) have been studied in various preclinical cancer models. Interestingly, non-selective β1/2-receptor antagonists like propranolol have shown to decrease tumor growth and migration of cancer cells [68, 70, 72, 176]. However, studies with β1-selective antagonists like atenolol did not demonstrate similar tumor inhibitory effects [68, 70, 177]. These studies highlight the crucial role played by β2-adrenergic signaling in tumorigenesis. β2-Adrenergic receptors are present both on the tumor cells and immune cells and abrogation of the signaling pathway via non-selective β-blockers is able to reverse the pro-tumorigenic cascade [39].

An “acute stress” is considered to be a single event that lasts minutes to hours, such as exposure to predator, which may activate the sympathetic “fight or flight” response and is then resolved, thus allowing the animal to return to its resting state [178]. On the other hand, “chronic stress” lasts for an extended period of time with no resolution and is known to have suppressive effects on immunity. This detrimental effect on immune cell function is mainly because of long and sustained physiological response, resulting in increase in catecholamine and glucocorticoid hormones [178, 179]. In preclinical models, there are several ways to mimic chronic stress such as social isolation, fear-inducing stimuli or cold stress of housing temperature, over long period of time [180]. Chronic stress results in higher levels of tissue catecholamines and greater tumor growth as seen in an orthotopic ovarian cancer mouse model. Increased NE primarily activates the tumor cell cyclic AMP (cAMP)-protein kinase A (PKA) signaling pathway by β2-receptor activation on ovarian cancer cells to trigger an increase in the expression of the vascular endothelial growth factor (VEGF) gene, resulting in enhanced tumor vascularization and aggressive growth and spread of malignant cells. Propranolol was shown to inhibit the β-receptor-dependent activation [181]. Choy et al. showed that the breast cancer cell line MDA-MB231, both primary and brain metastases cells, expressed the β2-adrenergic receptor. Treatment with propranolol resulted in development of brain metastases at a significantly decreased rate (*p* < 0.001) compared to the control cells [182].

Stress in an orthotopic mouse model of breast cancer resulted in increased infiltration with macrophages in the primary tumor parenchyma, with M2 differentiation, and thus induced a prometastatic gene expression. Propranolol treatment reversed the stress-induced macrophage infiltration and inhibited tumor spread. Macrophage suppression via CSF-1 receptor kinase inhibitor also inhibited formation of distant metastases, thus showing that β-adrenergic signaling has been shown to induce a pro-metastatic environment in primary breast cancer [72]. Another study has shown that chronic stress leads to increased β-adrenergic signaling resulting in creation of a “pro-metastatic” niche in the lungs, favoring colonization with circulating breast cancer cells. Increased β-adrenergic signaling upregulates the expression of CCL2 in the pulmonary stromal cells and CCR2 in monocytes/macrophages, thus recruiting macrophages into the pre-metastatic lung. Propranolol suppressed this stress-induced lung metastasis [183]. Nagaraja et al. showed that increased β-adrenergic signaling promoted pro-tumor and pro-metastatic cancer-associated fibroblasts (CAF) via induction of inhibin β A (INHBA) production by cancer cells after NE stimulation, resulting in increased collagen deposition in tumors. This resulted in potentiation of migration and invasion of cancer cells and decreased access of chemotherapy/immunotherapy along with cytotoxic T-cells. These effects were abrogated by propranolol by decrease in α-smooth muscle actin (SMA) levels and thus reduced expression of CAF markers and INHBA [184].

β2-Adrenergic receptor (AR) is the primary subtype expressed on immune cells (including T-cells, B-cells, dendritic cells, and macrophages) [185, 186]. Our group showed that chronic stress via NE production acts on the β2 adrenergic receptors present on the CD8^+^ T-cells in a 4T1 murine breast cancer model, thus limiting anti-tumor immunity. This effect can be blocked by treatment with propranolol or via using β2-receptor knockout mice, thus resulting in the restoration of anti-tumor immunity as evidenced by increased expression of markers of effector function (T-bet, IFNγ, and GzmB) and a significantly increased ratio of IFNγ^+^ CD8^+^: Treg cells indicative of an inflammatory tumor microenvironment (TME). Since 4T1 cells do not express any β-receptors, the decrease in tumor growth seen with propranolol is attributed to the improved anti-tumor immune response induced with propranolol treatment. In addition, CD8^+^T cells in propranolol-treated mice also expressed lower levels of PD-1, which also improves the responses of these tumors to anti-programmed death receptor-1 (PD-1) checkpoint blockade therapy [47]. We also found that one mechanism by which β2-AR signaling can inhibit CD8^+^T cell activation is by metabolic reprogramming (glycolysis and mitochondrial oxidative phosphorylation) which is observed when the immune cells are activated. Murine CD8^+^T cells treated with pan β-AR agonist, isoproterenol (ISO), have reduced expression of glucose transporter 1 and decreased glucose uptake and glycolysis, compared to cells activated in the absence of ISO. The effect of ISO was specifically dependent upon β2-AR, since it was not observed in ADRB2^−^/^−^ CD8^+^T cells and was also blocked by propranolol [41]. In addition to the impact of β2-AR signaling pathway on CD8^+^T cells, our group showed that chronic stress acting via β2-receptor signaling results in promotion of proliferation and survival of myeloid derived suppressor cells (MDSC) in a murine breast cancer model [46], leading to suppression of antitumor immune response. The authors also show that inhibiting β2-AR signaling pathway by non-selective blocker, propranolol; or using β2-AR^−^/^−^ MDSC could decrease MDSC accumulation, their immunosuppressive function, and thus increase efficacy of antitumor immune response and inhibition of tumor growth.

Findings from these preclinical models of chronic stress are applicable to the clinical setting when patients are diagnosed with cancer, as the physical and psychological symptoms from cancer diagnosis along with cancer treatment is considered a long-standing stressful situation for these patients.

### Clinical studies using pharmacological blockade

There have been several studies in literature so far reporting on the role of impeding the stress associated β-AR signaling pathway to improve cancer-related outcomes. Here, we cite some examples of retrospective studies and prospective clinical trials in breast cancer highlighting the importance of targeting this pathway. Barron et al. prospectively collected data from the National Cancer Registry Ireland (NCRI) to identify women diagnosed with stage I–IV breast cancer. Patients were stratified into those taking propranolol or atenolol or no β-blockers before breast cancer diagnosis for non-oncologic reasons. Breast cancer patients on propranolol were significantly less likely to present with advanced stage tumor (T4 or N2/N3/M1) and furthermore had a lower breast cancer-specific mortality (HR 0.19, 95% CI 0.06–0.60) when compared to matched non-users. However, on the other hand, there was no difference between advanced stage tumor presentation or breast cancer mortality among patients on β1-selective blocker atenolol compared to matched non-users. This clearly demonstrates and concurs with the preclinical findings that the effect of propranolol are the result of β2-AR antagonism [187].

A retrospective study by Choy et al. showed that for stage II breast cancer patients, treatment with β-blocker perioperatively significantly reduced the risk of postoperative recurrence or distant metastasis (HR 0.51; 95% CI 0.23–0.97, *p* = 0.041) [182]. In another retrospective cohort study, non-selective β-blocker (but not selective β-blocker) was shown to decrease the tumor proliferative index (Ki67) by 66% (*p* < 0.001) in early-stage breast cancer. In addition, on prospective analysis of one patient, propranolol treatment resulted in reduction in Ki67 by 23% between pretreatment and posttreatment biopsy [188]. These findings could be explained by the fact that psychological stress is increased following a diagnosis of breast cancer, especially, at the time of surgery. A prospective study randomized breast cancer patients to control and propranolol groups after undergoing modified radical mastectomy until day 3 postsurgery and peripheral blood samples were collected until 7 days postsurgery. The study showed that surgery results in an increase in the number of immunosuppressive T-regulatory cells (Treg), thus showing that chronic stress is immunosuppressive and could contribute to future recurrence; however, the Treg increase was abrogated with propranolol treatment [189]. A recent prospective clinical trial including all breast cancer subtypes showed that preoperative β-blocker downregulated biomarkers of invasive potential and improved biomarkers of cellular immune response within the breast tumor. Interestingly, the study showed that patients with clinical evidence of disease response (lowered heart rate and blood pressure) showed elevated tumor infiltration of CD68^+^ macrophages and CD8^+^T cells [190]. These studies have analyzed and confirmed the importance of β-blockade on recurrence and proliferation markers in early stage breast cancer patients in the pre- and perioperative setting.

The impact of β-AR blockade on cancer outcomes appears to be independent of breast cancer subtype with clinical responses in TNBC and Her2-positive breast cancer, and its role in hormone-receptor positive breast cancer is still being investigated. In a retrospective study of 800 postmenopausal TNBC patients, β-blocker intake (for non-oncologic reasons) improved recurrence-free survival (RFS) in women with TNBC and also reduced the risk of metastases [191]. Another study shows that trastuzumab-resistance-dependent PI3K/AKT/mTOR pathway is controlled by catecholamine-induced β2-AR pathway. Studies have shown that propranolol treatment not only enhances the activity of trastuzumab but also resensitizes the resistant cells to trastuzumab. Retrospective case-control study showed improved PFS and OS in the population on concurrent propranolol, trastuzumab, and chemotherapy treatment compared to only trastuzumab and chemotherapy in Her2-overexpressing metastatic breast cancer [192]. Table 1 shows examples of other studies which report the positive impact of beta-blocker use specifically in breast cancer patients on recurrence and mortality [35, 193–195].

**Table 1:** Overview of some retrospective studies and meta-analysis reporting outcomes with beta-blocker use in breast cancer

Study	Population	Timing of β-blocker use	Breast cancer outcomes
Childers et al.	Meta-analysis of 7 studies: systematic review using Cochrane library and PubMed	-	No statistically significant reduction in breast cancer recurrence. Significant reduction in breast cancer death (HR 0.50; 95% CI 0.32–0.80). No significant effect of β-blockers on all-cause mortality (HR, 1.02; 95% CI, 0.75–1.37) [35]
Ganz et al.	LACE (Life After Cancer Epidemiology) cohort: Early stage invasive breast cancer	β-blocker use in the year prior to or after breast cancer diagnosis	β-blocker use was associated with lower hazard of recurrence (HR 0.86) and cause-specific mortality (HR 0.76) but not statistically significant [193]
Melhem-Bretrandt et al.	Retrospective analysis in breast cancer patients receiving neoadjuvant chemotherapy	β-blocker use at the start of neoadjuvant chemotherapy	β-blocker intake was associated with a significantly better RFS (HR 0.52, 95% CI 0.31 to 0.88) but not OS. Among patients with TNBC, β-blocker intake was associated with improved RFS (HR 0.30; 95% CI 0.10 to 0.87; *p* = 0.027) but not OS. [194]
Powe et al.	Retrospective study in patients with operable breast cancer	β-blocker use prior to cancer diagnosis	β-blocker-treated patients showed a significant reduction in metastasis development (*p* = 0.026), tumor recurrence (*p* = 0.001), and longer disease-free interval (*p* = 0.01). In addition, there was a 57% reduced risk of metastasis (hazards ratio = 0.430; 95% CI = 0.200–0.926, *p* = 0.031), and a 71% reduction in breast cancer mortality after 10 years (Hazards ratio = 0.291; 95% CI = 0.119–0.715, *p* = 0.007) [195].

Based on the above studies, besides the standard use of β-blockers in medical indications, like cardiac arrhythmias, hypertension, infantile hemangioma, thyrotoxicosis, tremors, migraine prophylaxis, cluster or tension headache, β-blockers have emerged as a significant repurposing agent in oncology due to its ability to abrogate the adrenergic signaling pathway via its anti-proliferative, anti-angiogenic, anti-lymphangiogenic, pro-apoptotic, and immunomodulating effects. There is some preclinical data for anti-cancer efficacy for carvedilol and atenolol [196–198]; however, most of the preclinical studies have used propranolol. In clinical practice, the frequently prescribed non-selective β-blockers are carvedilol, labetalol, nadolol, and sotalol for non-oncologic reasons as discussed above. New preclinical studies in the future should consider utilizing the more commonly prescribed β-blockers, in addition to propranolol so the findings could be translated into real-world practice. The β-adrenergic affinity for all these agents vary and new research and future studies will be needed to show if breast cancer subtypes with different expression levels will be sensitive to different β-blockers.

## Future directions

Preclinical studies as discussed above show that stress can act not only on the tumor and stromal cells but also on the immune cells creating an immunosuppressive milieu which promotes tumor growth. Inhibition of β2-AR signaling is an effective mechanism to overcome the detrimental impact of chronic stress in cancer. There has been increasing interest in bench-to-bedside translation of this important finding which potentially may provide a promising avenue for managing stress among breast cancer patients.

Retrospective studies have several limitations which include presence of different comorbidities, type, dose, and duration of use of different β-blockers for which it is difficult to control the retrospective data. Hence, there is a need to investigate their use in prospective clinical trials in addition to standard chemotherapeutic and immunotherapeutic modalities to improve cancer outcomes. Several clinical trials in breast cancer are evaluating this crucial role of propranolol in early stage and metastatic breast cancer (NCT01847001, NCT02013492). Both studies being performed are expanding on the previous laboratory data which demonstrates that propranolol inhibits breast cancer progression. NCT01847001 is investigating the combination of propranolol with chemotherapy in influencing biomarkers, like Ki67, tumor density and change in stress score levels in the neoadjuvant setting in early-stage breast cancer. NCT02013492 is studying the role of propranolol on the tumor microenvironment and host immune system, in addition to survival outcomes in the metastatic setting.

## Conclusion

Cancer affects patients and their families in many ways, increasing stressful conditions, physically, mentally, and emotionally. Multiple studies have shown that stress promotes breast cancer growth. To reduce cancer-related stress, patients can use several modalities, mainly pharmacological or non-pharmacological. The use of CAM techniques in assisting patients to enhance their quality of life has been on a rise. There is an important opportunity for transdisciplinary research to integrate findings from preclinical and clinical studies on anti-tumor immunity to understand the potential of common non-pharmacological stress reduction strategies (such as mindfulness and exercise) to alter the relationship between stress and cancer progression. Use of β-blockers to inhibit the elevated β2-adrenergic receptor signaling observed in cancer patients experiencing stress has been shown to decrease tumor progression both in preclinical and clinical studies, and offers a relatively inexpensive and safe option to combine with standard of care cancer treatments. In the future, if there is a way to quantify stress with a stress signature, it may provide us with a biomarker to streamtailor the targeted use of non-pharmacologic and pharmacologic strategies to this population.
